# The Role of Wild-Type p53 in Cisplatin-Induced Chk2 Phosphorylation and the Inhibition of Platinum Resistance with a Chk2 Inhibitor

**DOI:** 10.1155/2011/715469

**Published:** 2010-12-01

**Authors:** Xiaobing Liang, Yi Guo, William Douglas Figg, Antonio Tito Fojo, Michael D. Mueller, Jing Jie Yu

**Affiliations:** ^1^Department of Biochemistry, School of Medicine, Department of Basic Pharmaceutical Sciences, School of Pharmacy, and Mary Babb Randolph Cancer Center, Robert C. Byrd Health Sciences Center, West Virginia University, Morgantown, WV 26506, USA; ^2^Molecular Pharmacology Section, Medical Oncology Branch and Affiliates, National Cancer Institute, National Institutes of Health, Bethesda, MD 20892, USA; ^3^Experimental Therapeutics Section, Medical Oncology Branch and Affiliates, National Cancer Institute, National Institutes of Health, Bethesda, MD 20892, USA

## Abstract

The major obstacle in platinum chemotherapy is the repair of platinum-damaged DNA that results in increased resistance, reduced apoptosis, and finally treatment failure. Our research goal is to determine and block the mechanisms of platinum resistance. Our recent studies demonstrate that several kinases in the DNA-repair pathway are activated after cells are exposed to cisplatin. These include ATM, p53, and Chk2. The increased Chk2 phosphorylation is modulated by p53 in a wild-type p53 model. Overexpression of p53 by cDNA transfection in wt-p53 (but not p53 deficient) cells doubled the amount of Chk2 phosphorylation 48 hours after cisplatin treatment. p53 knockdown by specific siRNA greatly reduced Chk2 phosphorylation. We conclude that wild-type p53, in response to cisplatin stimulation, plays a role in the upstream regulation of Chk2 phosphorylation at Thr-68. Cells without normal p53 function survive via an alternative pathway in response to the exogenous influence of cisplatin. We strongly suggest that it is very important to include the p53 mutational status in any p53 involved studies due to the functional differentiation of wt p53 and p53 mutant. Inhibition of Chk2 pathway with a Chk2 inhibitor (C3742) increased cisplatin efficacy, especially those with defective p53. Our findings suggest that inhibition of platinum resistance can be achieved with a small-molecule inhibitor of Chk2, thus improving the therapeutic indices for platinum chemotherapy.

## 1. Introduction

DNA targeting agents for cancer treatment are among the most common treatments to fight cancer. However, side effects and drug resistance of chemotherapy are a major clinical problem, seriously affecting both the life quality of patients and the outcome of treatment. Studies show that cells treated with genotoxic agents swiftly respond by activating DNA-damage checkpoint response. This prompts the repair of DNA lesions while transiently slowing down replication, or it elicits an apoptotic program in case of massive or irreparable lesions to the DNA. Two primary pathways are initiated in response to DNA damage. One is mediated by the ATM-Chk2 axis and the other, via the ATR-Chk1 axis. The ATM/Chk2 pathway responds primarily to DNA double-strand breaks, whereas the ATR-Chk1 pathway mainly responds to replication-associated DNA lesions [1, 2].

The transcription factor p53, a DNA-binding protein containing DNA-binding, oligomerization, and transcriptional activation domains [3–6], plays a key role in the DNA damage response to genotoxic stress by binding directly to the promoters of target genes and altering the rate at which they are transcribed [7, 8]. Under normal conditions wild-type p53 is maintained at a low level due to the extremely short half-life of the polypeptide, and in a largely inactive state that is inefficient for its function [7–10]. In response to genotoxic stress, wt p53 proteins form a tetramer that binds DNA for exerting its transactivate function [6, 11]. This will lead to the activation of numerous genes that cause growth arrest and apoptosis [7–11]. 

Checkpoint kinase 2 (Chk2), a serine/threonine kinase and encoded protein, contains a forkhead-associated protein interaction domain. It lies at the heart of the DNA damage/repair pathway and is responsible for the maintenance of mammalian genomic integrity. Following exposure to ionizing radiation, Chk2 is rapidly activated by ATM and DNA-PK (DNA-dependent protein kinase) via phosphorylation at Thr^68^ of Chk2, causing homodimerization and subsequent *trans*-activating autophosphorylations at Thr^383^ and Thr^387^ and *cis*-phosphorylation at Ser^516^ [1, 2, 12]. Chk2 activation occurs in an ATM-independent manner in response to UV radiation or stalled DNA replication [13]. In turn, activated Chk2 phosphorylates downstream substrates, including Cdc25A on serine^123^ and Cdc25C on Ser^215^, and inhibits Cdc25C phosphatase, preventing entry into mitosis, leading to cell cycle arrest in G1 phase. This protein also interacts with and phosphorylates BRCA1, allowing it to restore survival after DNA damage [2, 12, 14, 15]. 

Recent studies suggest that Chk2 inhibition in combination with genotoxic agents might have therapeutic value. Inhibition of Chk2 expression reduces DNA damage-induced cell cycle checkpoints and enhances apoptosis in p53-defective HEK-293 cells [12, 16]. Chk2 inhibition also increases the level of mitotic catastrophe and sensitizes proliferating cells to doxorubicin-induced apoptosis [17]. Molecular or genetic targeting of Chk2 prevents the release of survivin from mitochondria and enhances DNA damage-induced tumor cell apoptosis, thus inhibiting *in vivo* growth of resistant tumors, providing a rational approach for treating these tumors [18]. In addition to augmenting the effect of cytotoxic drugs, Chk2 inhibitors may elicit radio- or chemoprotection of normal tissue via abrogation of p53-dependent apoptosis [19].

This investigation focused on the cisplatin-induced activation and regulation of Chk2 and further defines the relationship between two central mediators, p53 and Chk2, of the DNA damage/repair signaling pathway. Our results suggest that, in specific conditions, Chk2 activation at Thr^68^ phosphorylation is regulated by p53 in response to cisplatin treatment in wt p53-contain cells, but not in p53-deficient cells, of human ovarian cancer. Using a Chk2 inhibitor to block this cellular pathway greatly enhanced the efficacy of cisplatin in cancer chemotherapy.

## 2. Materials and Methods

### 2.1. Cell Culture and Drug Treatment

Human ovarian cancer cell lines A2780, Caov-3, and SKOV-3 were used in the current investigation. Cells were cultured in monolayer using RPMI 1640 media supplemented with 10% (v/v) fetal calf serum, 2 mM L-glutamine, 0.2 units/mL human insulin, 50 units/mL penicillin, and 50 mg/mL streptomycin (Life Technologies, Inc., Gaithersburg, MD, USA). Cells were grown in logarithmic growth at 37°C in a humidified atmosphere consisting of 5% CO_2_, 95% air. Cells were routinely recovered from frozen stocks and allowed to grow until 80% confluence. For all experiments, cells were plated the day before exposure to treatment. 

Cisplatin (CDDP) (Sigma-Aldrich, St. Louis, MO) was prepared fresh daily by first dissolving it in phosphate-buffered saline (PBS), without Ca^++^ or Mg^++^, at a concentration of 1 mg per ml, and then further diluting it into prewarmed media to achieve the IC_50_ doses of 5-day survival (3 *μ*M for A2780, 10 *μ*M for Caov-3, and 75 *μ*M for SKOV-3 cells, resp.). For the time-course studies, cells were allowed to grow for 24 hr and treated with cisplatin for 1 hr at IC_50_ doses. At the end of 1 hr exposure to cisplatin, cells were washed twice with PBS and then incubated with fresh drug-free media for specifically required period of time.

### 2.2. Protein Extraction and Western Blotting

Treated and untreated cells were extracted with whole cell lysis buffer (20 mM Tris-HCl (pH 7.5), 150 mM NaCl, 1 mM Na_2_EDTA, 1 mM EGTA, 1% Triton, 2.5 mM sodium pyrophosphate, *β*-glycerophosphate, 1 mM Na_3_VO_4_, 1 mM PMSF, 10 *μ*g/mL leupeptin, 10 *μ*g/mL aprotinin, and 5 *μ*g/mL pepstatin) for 30 min before centrifugation (at 14,000 rpm for 30 min at 4°C). Supernatant was collected as whole cell lysate for Western blot analysis. Protein concentrations of extracts were determined by Bio-Rad Protein Assay kit (Bio-Rad, Hercules, CA) with bovine serum albumin as standard.

The whole cell lysates were separated on 12% SDS-polyacrylamide gels and transferred to PVDF membrane (BIO-RAD) using standard electrophoresis and electroblotting procedures. Prestained molecular weight markers were purchased from Invitrogen (Carlsbad, CA). To reduce nonspecific binding, blots were preincubated for 1 hr in a blocking buffer (5% nonfat dry milk, 1X TBS, and 0.1% Tween 20). Membranes were then incubated with primary antibodies overnight at 4°C. The primary antibodies applied were anti-ATM, anti-p-p53 phosphoserine-15, anti-p-p53 phosphoserine-20, anti-p53, anti-Chk2 phosphothreonine-68, anti-Chk2, anti-P48, and anti-P21. To demonstrate an equal loading of each sample, membranes were reprobed with *β*-actin using anti-*β*-actin antibody (Sigma). The signals of immunoreactive proteins were visualized using horseradish peroxidase-conjugated sheep antimouse or donkey antirabbit antibodies and enhanced by Chemiluminescence ECL detection system (Amersham, UK).

### 2.3. p53 Stable and Transient Transfection

A2780 and SKOV-3 cells were transfected with the plasmid pC53-SN3 or pCMV-Neo-Bam (provided by Dr. Bert Vogelstein) for the expression of wild-type human p53. Twenty-four hours after transfection, cells were treated with cisplatin at IC_50_ doses for 1 hr and then were continuously incubated with fresh media for indicated hours. Stable cell lines were established by selection of positive transfected clones that grew in media containing G418 prior to cisplatin treatment. Cell lysate was obtained by lysing the cells in buffer (20 mM HEPES (pH 7.0), 1 mM DTT, 1 mM MnCl_2_, 100 *μ*g/mL BSA, and 50 *μ*M leupeptin) and performed for Western blot analysis.

### 2.4. siRNA-Mediated p53 Silencing Assay

Small interfering RNAs (siRNAs) against p53 (SMARTpool p53) were purchased from Upstate Biotechnology (Lake Placid, NY). The siRNA Transfection Reagent Lipofectamine 2000 (Invitrogen) was used to transfect the siRNA into A2780 cells at a final concentration of 100 nM. A negative nonspecific siRNA was used as control. Twenty-four hours after transfection, cells were treated with cisplatin for 1 hr at IC_50_ dose. At the end of 1 hr exposure to cisplatin, cells were washed twice and then incubated with fresh drug-free media for indicated hours. The cells were harvested and protein level of p-Chk2, Chk2, p-p53, p53, and P21 were determined by western blotting.

### 2.5. Chk2 Transient Transfection

A2780 cells were transfected with the plasmid pEF-BOS-HA, pEF-BOS-HA-Chk2, or pEF-BOS-HA-Chk2D347A (provided by Dr. Jann N. Sarkaria) for control or the expression of wild-type human Chk2 or the expression of Chk2 kinase-dead point mutant. Twenty-four hours after transfection, cells were treated with cisplatin, and then were measured their expression levels.

### 2.6. Cytotoxicity Assay

A2780 or Caov-3 cells (around 1000 per well) were seeded in 96-well plates for 24 hrs and pretreated with different concentrations of Chk2 inhibitor II C3742 for 30 min. Subsequently, cells were treated with cisplatin at IC_50_ dose for 1 hr while continuously exposed to Chk2 inhibitor. After drug removal, cells were washed twice with PBS, and then incubated with fresh medium containing Chk2 inhibitor for 5 days. Cytotoxicity assay was performed following company's protocol of Cell Proliferation Kit I (Roche) and the complete solubilization of the purple formazan crystals was measured. Optical density was read at 600 nm to determine cell quantity. 

## 3. Results

### 3.1. Cisplatin Induced Phosphorylation of p53 and Chk2 in A2780 Cells

**Figure 1:**
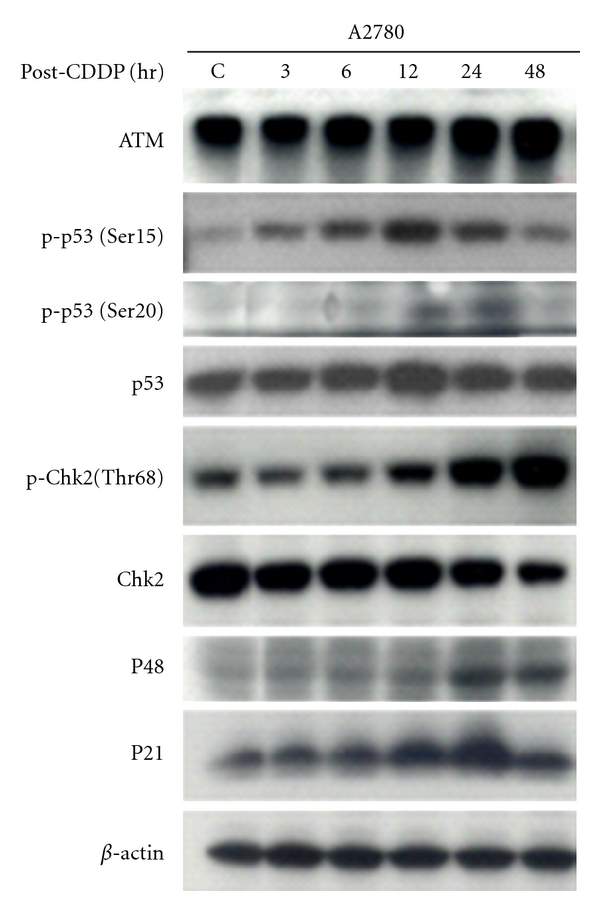
Cisplatin-induced DNA damage response in ovarian cancer A2780 cells. A2780 cells were treated with 3 *μ*M cisplatin (CDDP) for 1 hr. Cells were then washed to remove cisplatin and reincubated for indicated hours in a replacement of fresh drug-free media. Cells were harvested, and proteins were extracted, separated on SDS-PAGE gel, transferred onto PVDF membrane, and then probed with antibodies of anti-ATM, anti-p-p53 (Ser15 & Ser20), anti-p53, anti-p-Chk2 (Thr68), anti-Chk2, anti-P48, anti-P21 or anti-*β*-Actin. *β*-Actin served as a control.

Human ovarian cancer A2780 cells were analyzed for p53 mutations within exons 4 through 9 and classified to p53 wild-type cell line (data not shown). A2780 cells were exposed to Cisplatin at 3 *μ*M (IC_50_) for 1 h, and Western blotting was performed to analyze the levels of selected proteins of DNA damage response pathway. We observed increased protein level of ATM, p53, P48, and P21 in a time-dependent manner (Figure 1). At 12 h after cisplatin treatment, the p53 level is highly accumulated, accompanied by p53 phosphorylation. After drug treatment, these cells demonstrated cisplatin-induced p53 phosphorylation at serine 15 and serine 20 and Chk2 phosphorylation at Thr-68. Further, the greatest increase in p53 phosphorylation induced by cisplatin occurred 12 hours before the primary increase in Chk2 phosphorylation, hinting that p53 activation occurs before Chk2. In addition, we observed that cisplatin-mediated activation of p53 resulted in activation of downstream proteins P48 and P21 (Figure 1). 

### 3.2. Overexpression of p53 in wt p53-Contain Cells Increased Chk2 Phosphorylation

**Figure 2:**
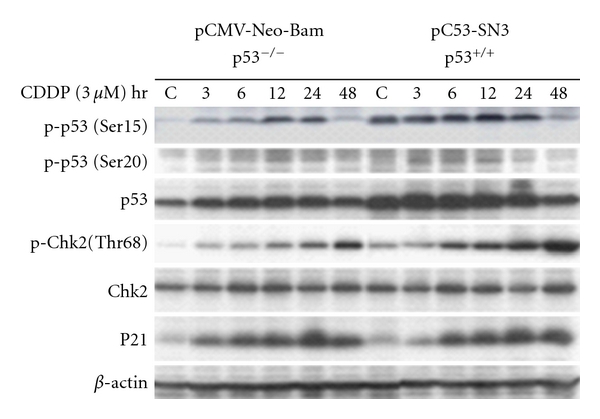
Overexpression of p53 by p53 transfection in A2780 cells. A2780 cells were transfected with the plasmid pC53-SN3 or pCMV-Neo-Bam for overexpression of wild-type human p53. Twenty-four hours after transfection, cells were treated with cisplatin (3 *μ*M) for 1 hr and then were continuously incubated with fresh media for indicated hours. Cell lysate was obtained by lysing the cells in lysate buffer and performed for Western blot analysis with antibodies of anti-p-p53(Ser15), anti-p-p53(Ser20), anti-p53, anti-p-Chk2(Thr68), anti-Chk2, anti-P21, or anti-*β*-Actin, respectively.

To determine the effect of p53 regulation on Chk2 activation, we transfected the p53 wild-type A2780 cells with plasmid pC53-SN3, which expresses the wild-type human p53, and pCMV-Neo-Bam, which is an empty vector of pC53-SN3. Twenty-four hours after transfection cells were treated with cisplatin at 3 *μ*M. Figure 2 shows that Chk2 phosphorylation is increased after p53 cDNA transfection. The overexpression of both p53 protein and phosphorylated p53 in these cells by cisplatin induction and cDNA transfection at least doubled the amount of observed Chk2 phosphorylation 48 hours after cisplatin stimulation.

### 3.3. Transfection of p53 in p53-Null Cells Failed to Alter Chk2 Activation

**Figure 3:**
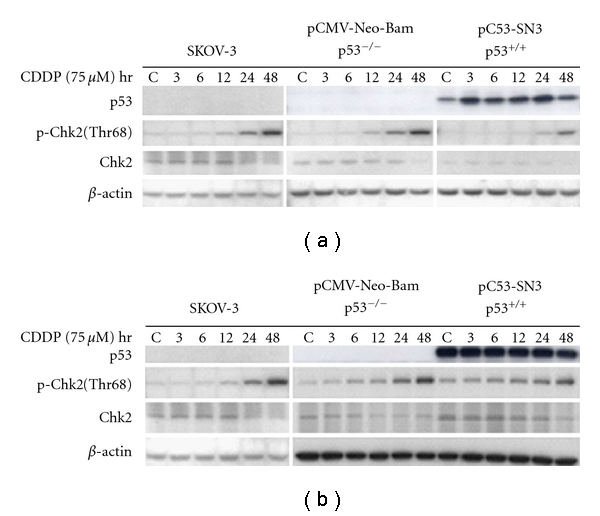
Transfection of p53 in p53-null SKOV-3 cells. Ovarian cancer SKOV-3 cells were transfected with the plasmid pC53-SN3 or pCMV-Neo-Bam for 24 hr: stable transfection (a) and transient transfection (b). Transfected cells were treated with cisplatin at IC_50_ dose (75 *μ*M) for 1 hr. The drug was removed by washing the cells with PBS. Cells were then incubated in fresh drug-free media for the indicated hours (3, 6, 12, 24, and 48) up to harvest. Western blot analysis was performed with indicated antibodies. Nontransfected cells were treated with cisplatin alone as control group.

We hypothesized that only the wt p53 phenotype represents the p53 functional system. Therefore, we transfected the plasmid of pC53-SN3 and of pCMV-Neo-Bam vector into SKOV3, p53-null cells. These cells were also exposed to cisplatin after a 24-hour transfection. Western analysis demonstrated no effect of Chk2 phosphorylation by p53 stable transfection (Figure 3(a)) or p53 transient transfection (Figure 3(b)). The similar expression pattern of phosphorylated Chk2 induced by cisplatin alone was seen in the three sample groups of control, negative, and positive transfected cells. This indicates that cells without wt p53, to survive, may adapt via an alternative pathway in response to cisplatin treatment. In other words, cisplatin-induced Chk2 activation may be regulated following an alternative pathway in the p53-null cells.

### 3.4. Inhibition of p53 by Specific siRNA Inhibited Chk2 Phosphorylation

**Figure 4:**
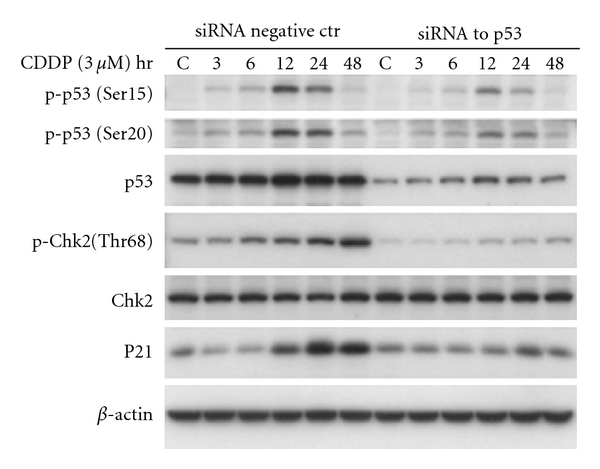
Inhibition of p53 by specific siRNA to p53 in A2780 cells. siRNA against p53 (SMARTpool p53) were transfected into A2780 cells using Lipofectamine 2000. A negative nonspecific siRNA was used as control. Twenty-four hours after transfection, cells were treated with cisplatin for 1 hr at 3 *μ*M concentration. At the end of 1 hr exposure to cisplatin, cells were washed and incubated with fresh media. The protein level of p-p53(Ser15), p-p53(Ser20), p53, p-Chk2(Thr68), Chk2, p21, and *β*-Actin were determined by Western blotting.

We performed p53 knockoff siRNA assay using small interfering RNA (siRNA) to silence the p53 then determine Chk2 expression in A2780 cells. The siRNA to human p53 contains 4 pooled SMART-selected siRNA duplexes with “UU” overhangs and a 5^'^ phosphate on the antisense strand (Upstate Biotechnology). As shown in Figure 4, in cells not treated with cisplatin, the siRNA to human p53 produced a decrease of phosphorylated Chk2, compared to the nonspecific siRNA-treated control. This decreased level may reflect a constitutive level of activated Chk2 68-phosphothreonine that is normally regulated by p53. Cells transfected by specific siRNA to p53 and treated with cisplatin resulted in a great reduction of p-Chk2 at Thr-68, suggesting that p53 modulates 68-threonine phosphorylation of Chk2.

### 3.5. Transfection of Chk2 in wt p53 Cells Did Not Alter p53 Level

**Figure 5:**
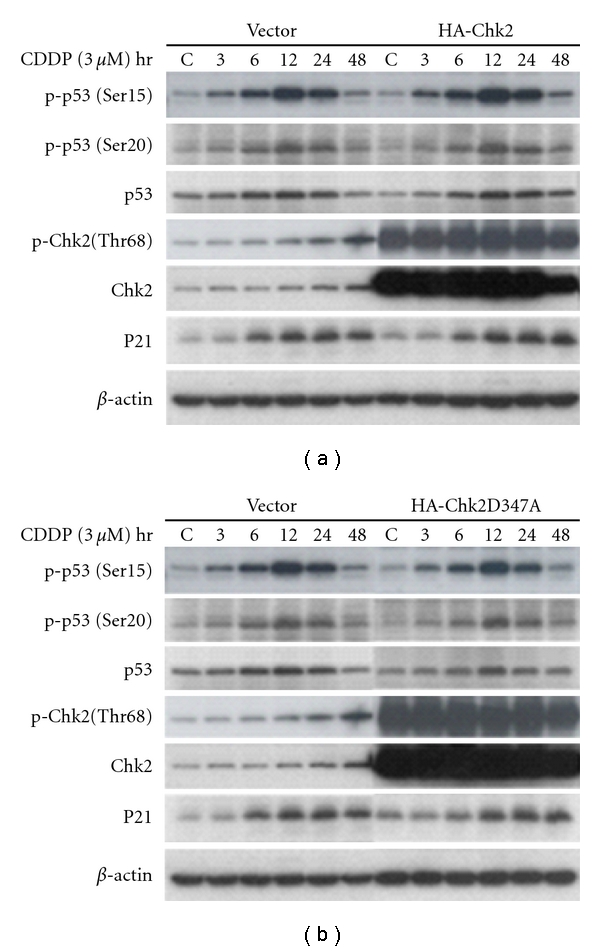
Overexpression of Chk2 by transfection of wild-type Chk2 or Chk2 dead allele in A2780 cells. A2780 cells were transfected with wild-type HA-Chk2 (a) or HA-Chk2 D347A (b) for overexpression of Chk2. Blank vectors were transfected as controls. Twenty-four hours after transfection, cells were treated with cisplatin (3 *μ*M) for 1 hr and then were incubated with fresh media. Cell lysate was obtained and performed for Western blot analysis with antibodies of anti-p-p53(Ser15), anti-p-p53(Ser20), anti-p53, anti-p-Chk2(Thr68), anti-Chk2, anti-p21, or anti-*β*-Actin, respectively.

Hypothetically, overexpression of Chk2 should not affect p53 level. To investigate this, we transfected the wt p53 A2780 cells with plasmid HA-Chk2, which expresses wild-type Chk2 (Figure 5(a)), and with HA-Chk2D347A, which is a kinase-dead Chk2 allele (Figure 5(b)). The empty vector was also transfected, respectively, as control. Cells were treated with cisplatin at IC_50_ dose 24 h after transfection. The results of Western blot analysis in these cells showed no effects of wild-type Chk2 (Figure 5(a)) or dead Chk2 (Figure 5(b)) on p53 protein or p53 phosphorylation (both Serine 15 and Serine 20). This suggests that Chk2 does not have a regulative effect on p53 in response to cisplatin treatment in ovarian cancer cells.

### 3.6. Application of Chk2 Inhibitor in Combination with Cisplatin Greatly Inhibited Cell Growth

**Figure 6:**
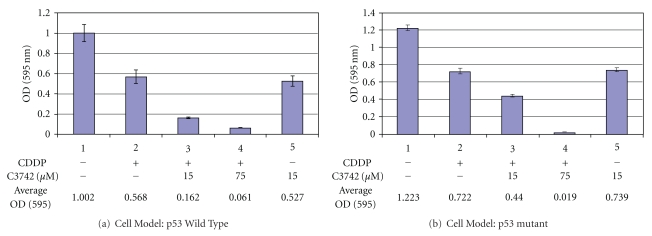
Application of Chk2 inhibitor in combination with cisplatin greatly inhibited cell growth. A2780 or Caov-3 cells (around 1000 per well) were seeded in 96-well plates for 24 hrs and pretreated with different concentrations of Chk2 inhibitor II C3742 for 30 min. Subsequently, cells were treated with cisplatin at IC_50_ dose for 1 hr while continuously exposed to Chk2 inhibitor. After drug removal, cells were washed twice with PBS, and then incubated with fresh medium containing Chk2 inhibitor for 5 days. Cytotoxicity assay was performed following company's protocol of Cell Proliferation Kit I (Roche) and the complete solubilization of the purple formazan crystals was measured. Optical density was read at 600 nm to determine cell quantity.

Cytotoxicity Assay was performed to validate our hypothesis that application of Chk2 inhibitor potentiates cisplatin efficacy in ovarian cancer treatment. Figure 6 shows that selective Chk2 Inhibitor (C3742, Sigma) greatly increased the cell-killing effect of cisplatin in wt p53 A2780 cells (Figure 6(a)). In combination with Chk2 inhibitor at 75 *μ*M, cisplatin produced about a 9-fold inhibition on cell growth, compared to cisplatin alone. This effect appears to be more significant (nearly 38-fold) in the mutant p53, Caov-3 cells (Figure 6(b)). We conclude that inhibition of Chk2 pathway with commercially available inhibitor will enhance the therapeutic indices of platinum compounds in the treatment of ovarian cancer, especially in those defective for p53 function.

## 4. Discussion

Disruption of the mechanisms that regulate checkpoint and apoptotic responses leads to genomic instability and the development of cancer [20]. It is in the best interest of the organism to prevent severely damaged cells from proliferating, either by halting them in a phase of cell cycle progression or by removing them entirely by apoptosis. The linear pathway of ATM-Chk2-p53 has been the dominant model and plays a central role in regulation of the cellular response to DNA damage, resulting in cell cycle arrest, DNA repair, or apoptosis depending on the cellular context and severity of DNA damage. In a well-studied example during double-strand-DNA-break damage, the ATM phosphatidylinositol 3-kinase-like serine/threonine protein kinase is activated by autophosphorylation at the Ser^1981^ site [21]. Activated ATM then phosphorylates several downstream substrates, including Chk2 and p53 kinases. When the cell enters the damage-repair process, phosphorylated p53 arrests the cell cycle by inducing the expression of cell cycle inhibitors such as p21 [21]. On the other hand, Chk2 contributes to p53 stabilization in response to IR [22], suggesting that Chk2 may, in this circumstance, be an upstream regulator of p53 [23]. In addition, the abundance of Chk2 mRNA was shown to be inversely related to p53 status in human gastric cancer, indicating the possibility that Chk2 is a downstream target of the p53 [24–26]. Other evidence supports the model of ATM-p53-Chk2, including: (a) Chk2 is not required for p53 responses in human cancer cells [27], (b) wild-type p53 suppresses mRNA and protein levels of Chk2 in human osteosarcoma Saos2 (p53 null) cells [28], (c) p53 mutations increase Chk2 expression in human gastric carcinoma [24], (d) Chk2 is dispensable for p53-mediated G_1_ arrest but is required for a latent p53-mediated apoptotic response [29], and (e) Chk2 expression is negatively regulated by functional p53, leading to a high level of expression in p53-deficient cancer cells [20, 30].

We observed that a cisplatin-induced increase in p53 phosphorylation preceded (by about 12 hours) the observed increase in Chk2 phosphorylation. Overexpression of p53 in studied cells by cDNA transfection doubled the amount of Chk2 phosphorylation 48 hours after cisplatin treatment, whereas p53 knockdown by p53 specific siRNA greatly reduced Chk2 phosphorylation. In contrast, overexpression of Chk2 by transfection of wild-type Chk2 in these cells did not show effects on p53 protein or on phosphorylated p53 (Ser15 and Ser20). These observations further define the relationship between the two central mediators, p53 and Chk2. Our data suggest that in specific conditions Chk2 activation by Thr^68^ phosphorylation is regulated by p53 in response to cisplatin treatment in wt p53-contain cells, but not in p53-deficient cells, of human ovarian cancer. In addition, transfection of p53 cDNA into p53-null SKOV-3 cells failed to alter Chk2 activation, suggesting that the Chk2 role occurs alternatively and p53 independently in p53 defective cells. We conclude that wild-type p53, in response to cisplatin stimulation, plays a role in the upstream regulation of Chk2 phosphorylation at Thr-68. Cells without normal p53 function survive via an alternative pathway in response to the exogenous influence of cisplatin. We strongly suggest that it is very important to include the p53 mutational status in any p53 involved studies due to the functional differentiation of wt p53 and p53 mutant.

One of the approaches in current cancer treatment is to identify drugs acting on specific cancer-relevant targets. Specific sensitization of tumor cells to the action of genotoxins would reduce the efficacious doses of genotoxins to be used in patients, diminishing the detrimental side effects of the drugs on normal tissues [31, 32]. The checkpoint kinase Chk2 is central to transmitting the DNA-damage signal. Parallel studies of the human Chk2 gene have highlighted its role as a candidate multiorgan tumor susceptibility gene rather than a tumor suppressor gene in the classical sense. Chk2 can prevent tumor progression by averting genomic instability through DNA repair. If DNA-targeting agent is combined with a Chk2 inhibitor, DNA repair will be impaired and tumor cells unable to arrest the accumulation of irreparable damaged DNA and consequently undergo apoptosis [12]. Chk2 negatively regulate a poorly defined type of death occurring during mitosis or resulting from failed mitosis (mitotic catastrophe) by activating G2/M arrest and preventing entry into mitosis, meaning that inhibition of Chk2 may sensitize tumor cells to chemotherapy-induced apoptosis [17]. In addition, Chk2 has been shown to induce release from the mitochondria of the antiapoptotic protein survivin, which is thought to inhibit apoptosis in cancer cells and might confer radiation resistance in human cancer cells [18]. Chk2 elicits distinct cellular outcomes in the presence or absence of extrinsic DNA damage within a specific cellular context, in particular p53 status [12]. 

As stated earlier, the substrates of Chk2 kinase impact both cell cycle checkpoint and apoptosis. In a p53-deficient tumor, Chk2 primarily affects cell cycle checkpoint. Thus, Chk2 inhibition would abrogate DNA damage-induced cell cycle arrest and sensitize the tumor to chemotherapeutic agents. In normal tissues, Chk2 acts as a proapoptotic effector; therefore, a Chk2 inhibitor would protect normal tissues but sensitize the tumor to chemotherapeutic agents [2, 12, 33]. Given the known role of Chk2 in ovarian cancer pathogenesis and the findings from our studies and by other groups, our goal is to block the Chk2-regulated DNA repair pathway using a Chk2 inhibitor to enhance therapeutic index for current cancer chemotherapy.
